# Barriers for Telemedicine Use Among Nonusers at the Beginning of the Pandemic

**DOI:** 10.1089/tmr.2021.0022

**Published:** 2021-10-05

**Authors:** Robyn B. Adams, Victoria R. Nelson, Bree E. Holtz

**Affiliations:** Department of Advertising and Public Relations, Michigan State University, East Lansing, Michigan, USA.

**Keywords:** telemedicine, COVID-19, patient–provider, technology

## Abstract

Telemedicine has garnered considerably more attention during the COVID-19 pandemic than in any time previously. However, before the beginning of the pandemic, many individuals had not accessed care in this manner. The purpose of this study was to understand the central reasons that individuals had not used telemedicine before the pandemic. Therefore, we conducted a convenience sample survey in March–April 2020, and 539 participants living in the United States answered questions about exploring their reasons for not having used telemedicine. Descriptive statistics and correlations were conducted to analyze the data. Two themes emerged from these data, including the importance of the patient–provider relationship and that access to technology was not the issue for this particular population. Although technology was not a barrier, many, specifically older participants, had concerns over privacy and security. As the world gains some control over the COVID-19 pandemic and medical appointments begin to return to a new normal, the implication for the continued use of telemedicine is still important to address as this will become a standard of care.

## Introduction

Telemedicine has a long history; however, it has gained immense popularity during the COVID-19 pandemic.^1^ Many providers and health systems started seeing patients for routine visits through telemedicine to lower the transmission of the virus and handle various lockdowns. However, before the pandemic, most individuals and health care providers had not experienced a telemedicine visit.^2^ Despite the increase of telemedicine services now, previous research reports that the uptake of these services has improved but only among specific populations (e.g., wealthier, urban, and adults).^3^ There are many reasons for this lack of experience with telemedicine including insurance coverage, reimbursement policies for the provider, not perceiving a need, not having the technology, not knowing how, that it was an option, and health care providers' reluctance.^4^

Past studies have examined the reasons why patients may not use telemedicine services suggesting that patients may have decided not to during the pandemic because of easy access to see their primary care provider (PCP) or not having the access to technology.^5,6^ A recent study examining PCPs' experience and perceptions of telemedicine found that physicians reported that telemedicine offered positive changes between provider–patient communication and new chances to improve the quality of care offered to patients.^6^ However, there is still a need to understand more about reservations patients may have with adopting telemedicine services.

More than a year has passed since COVID-19 was identified, and the question remains; once the virus has been contained and controlled, will telemedicine remain an option to receive health services? This study's objective was to identify the main barriers for nontelemedicine use by patients before the pandemic, and second, examine demographic variables related to nonuse to understand ways to improve telemedicine uptake among nonusers of these services. Based on the recent research finding that patients during the pandemic relied on their PCPs to use telemedicine, therefore, we will examine how nonusers' relationship with their PCPs, perceptions of quality of care, understanding of telemedicine, privacy concerns, and technology access influence their decision making. The information gained from this study can help encourage and support people who might be tentative to use the service when the threat of the global pandemic has largely passed. By addressing this study's objectives, we provide recommendations on improving the adoption of telemedicine.

## Methods

### Participants

Participants for this study were recruited through convenience sampling. An invitation to participate in the study was shared through social media and email listservs. To be eligible to participate, individuals had to be at least 18 years old and provide consent. The study was approved by the university's institutional review board.

### Procedure and measures

This cross-sectional online survey (Qualtrics) was developed through a review of the literature and modifying surveys used in the past regarding telemedicine use.^7,8^ For this study, we used only the participants who reported they had never had a telemedicine visit. The survey took ∼10–15 min to complete and was available from March 31 to April 20, 2020.

The survey included 23 items regarding reasons for not having used telemedicine (all items are listed in Table 2). Example statements of nonuse included, “I would get better care if I see my provider in-person,” “It easy to get to my provider,” and “I don't have technology for service” and the items were answered on a 5-point Likert-type scale, where 1 = strongly agree and 5 = strongly disagree. Demographic questions, including gender, race, education, income, insurance status, overall health status, having a PCP, and access to the internet were also asked.

**Table 2.** Survey Items, Means, and Standard Deviations

Survey item	Mean (SD)
General COVID-19	
I would most likely use telemedicine now because of coronavirus (COVID-19)	1.78 (0.83)
PCP	
It is easy to get into my PCP	2.46 (1.08)
I am concerned that my PCP would not get my visit information	3.16 (1.09)
I would get better care if I see my provider in person	2.62 (1.00)
I have not had a need to see a health care provider	2.79 (1.24)
I am worried about the continuity of care (i.e., I do not see the same provider every time)	2.81 (1.14)
Quality and experience of care	
I am worried about the quality of care I would receive	3.03 (1.07)
I prefer to going to walk-in clinics	3.15 (1.20)
I think I would get better care in person	2.46 (0.95)
I am worried about the ability to communicate adequately with the health care provider	3.18 (1.16)
I worry about the quality of communication with a provider using telemedicine	3.22 (1.12)
I am worried about the accuracy of the information from the telemedicine health care provider	3.32 (1.06)
I am worried that the health care provider will not be sensitive to my needs	3.45 (1.07)
I think it would take longer to have a visit over telemedicine than in person	4.07 (0.80)
Lack of understanding	
I am unsure whether my insurance covers these visits	3.10 (1.29)
I do not know how to find telemedicine services	3.19 (1.22)
I do not know how to get telemedicine care	3.28 (1.25)
Privacy	
I am worried about the confidentiality of my private information being exchanged through telemedicine	3.61 (1.12)
I am not technology savvy enough to use telemedicine services	4.44 (0.81)
Technology	
I am worried that I will have difficulty seeing the health care provider over telemedicine	4.25 (0.84)
I am worried that I will have difficulty hearing the health care provider over telemedicine	4.29 (0.85)
I do not have very good internet	4.30 (0.85)
I do not have the technology needed for telemedicine services	4.35 (0.78)

1 = strongly agree; 2 = agree; 3 = neutral; 4 = disagree; 5 = strongly disagree.

### Statistical analysis

Descriptive statistics were used to describe the population and rank the reasons for nonuse of telemedicine. In addition, Pearsons correlations to assess the relationship between demographic factors and the reasons for nonuse were computed. All statistical analyses were done using SPSS (v.27).

## Results

A total of 978 participants completed the survey. Of those responses, 539 participants (55.11%) had not used telemedicine. Among nonusers of telemedicine, 92.6% (*n* = 499) of participants were female, 87% (*n* = 469) identified as White, 84.6% (*n* = 456) had a PCP, 96.6% (*n* = 520) had some form of medical insurance, and >91% (*n* = 495) had access to high-speed internet (see Table 1 for full demographics).

**Table 1.** Demographics

	Total, *N* (%)
	Nonusers of telemedicine
Gender	
Female	499 (92.6)
Male	27 (5)
Gender nonconforming or transgender	1 (0.2)
Prefer not to answer	3 (0.6)
Year born	
1930–1950	5 (0.93)
1951–1970	136 (25.23)
1971–1990	300 (55.66)
1991–2011	35 (6.49)
Prefer not to answer	63 (11.7)
Income	
<$10,000	2 (0.4)
$10,000–19,999	5 (0.9)
$20,000–29,999	9 (1.7)
$30,000–39,999	17 (3.2)
$40,000–49,999	15 (2.8)
$50,000–59,999	30 (5.6)
$60,000–69,999	28 (5.2)
$70,000–79,999	37 (6.9)
$80,000–89,999	32 (5.9)
$90,000–99,999	34 (6.3)
$100,000–149,999	125 (23.2)
$150,000+	136 (25.2)
Prefer not to answer	59 (10.9)
Education	
Grades 1–12 (no diploma)	3 (0.6)
High school diploma	2 (0.4)
GED or alternative credential	2 (0.2)
Some college (no degree, some college credit, 1 or more years)	31 (5.8)
Associate's degree (e.g., AA, AS)	16 (3)
Bachelor's degree (e.g., BA, BS)	207 (38.4)
Master's degree (e.g., MS, MEng, MEd, MSW, MBA)	159 (29.5)
Professional degree beyond bachelor's degree (e.g., MD, DDS, DVM, LLB, JD)	47 (8.7)
Doctorate degree (e.g., PhD, EdD)	63 (11.7)
Employment status	
Employed for wages	394 (73.1)
Self-employed	36 (6.7)
Out of work and looking	11 (2)
Out of work but not currently looking	8 (1.5)
homemaker	25 (4.6)
Student	15 (2.8)
Retired	29 (5.4)
Unable to work	2 (0.4)
Other	8 (1.5)
Insurance status	
Insurance through current employer or former employer or union	414 (76.8)
Insurance purchased directly from an insurance company	30 (5.6)
Medicare	17 (3.2)
Medicaid	9 (1.7)
TRICARE or other military health care	2 (0.4)
Other	48 (8.9)
No health insurance	7 (1.3)
Race	
White	469 (87)
Black or African American	16 (3)
American Indian, Alaskan Native, Native Hawaiian	1 (0.2)
Asian Indian	4 (0.7)
Asian	5 (0.9)
Pacific Islander	1 (0.2)
Other	3 (0.6)
Hispanic	
No, I am not Hispanic, Latino, or Spanish origin	474 (87.9)
Mexican	9 (1.7)
Puerto Rican	3 (0.6)
Another Hispanic	5 (0.9)
Overall health status	
Mean (SD)	2.19 (0.80)
Have a PCP	
Yes	469 (74.4)
No	78 (12.4)
Access to internet^a^	
Cellular data plan for a smart phone or other mobile device	378 (70.2)
Broadband (high speed) internet service such as cable, fiber optic, or DSL service	495 (91.8)
Satellite internet service	16 (3)
Technology^a^	
Desktop computer	167 (26.5)
Laptop	490 (77.8)
Tablet	346 (54.9)
E-reader	182 (28.9)
Mobile phone	526 (83.5)
Smartwatch (apple watch, Fitbit, etc.)	154 (24.4)

^a^ Each category is out of 100%.

DSL, digital subscriber line; GED, General Education Development; PCP, primary care provider; SD, standard deviation.

When ranking the reasons for the nonuse of telemedicine services among nonusers, two primary themes emerged. The first was participants having a close relationship with their PCP. Specifically, many of the individuals stated that they found it easy to get in to see their physician (mean = 2.45, standard deviation [SD] = 1.08), they felt they got better care in-person (mean = 2.62, SD = 1.00), and they worried about the continuity of care if using telemedicine (mean = 2.81, SD = 1.14). However, the impact of COVID-19 affected the participants' willingness to consider utilizing telemedicine services in the future (mean = 1.78, SD = 0.83). The second theme demonstrated that for this particular population, technology access (mean = 4.44, SD = 0.81) and literacy-related issues (e.g., tech savvy; mean = 4.44, SD = 0.81) were not barriers. That technology access were not barriers is demonstrated by the significant relationships with demographic factors such as educational attainment (*r* = −0.12, *p* ≤ 0.001) and income (*r* = 0.09, *p* = 0.04; see Table 2 for full survey items, means, and SDs and Table 3 for correlation data.)

**Table 3.** Correlations Between Demographic Information and Reasons for Nonuse of Telemedicine

Measure	Gender	Age	Income	Degree	Employment status
I prefer going to walk-in clinics	0.02 (0.62)	−0.10 (0.03)^*^	0.04 (0.42)	−0.03 (0.56)	0.02 (0.59)
I am unsure whether my insurance covers these visits	−0.06 (0.14)	0.08 (0.09)	0.16 (<0.001)^**^	0.07 (0.10)	−0.02 (0.66)
I am worried about the ability to communicate adequately with the health care provider	−0.06 (0.14)	−0.11 (0.02)^*^	0.08 (0.09)	0.02 (0.71)	0.04 (0.4)
I think I would get better care in person	−0.07 (0.12)	−0.09 (0.05)^*^	0.03 (0.59)	−0.02 (0.73)	0.02 (0.66)
I do not have very good internet	−0.02 (0.71)	0.02 (0.63)	0.09 (0.05)^*^	0.09 (0.03)^*^	−0.19 (<0.001)^**^
I am not technologically savvy enough to use telemedicine services	−0.04 (0.36)	0.11 (0.02)^*^	0.08 (0.09)	0.16 (<0.001)^**^	−0.13 (<0.001)^**^
I am worried that I will have difficulty hearing the health care provider over telemedicine	0.01 (0.75)	0.04 (0.45)	0.11 (0.01)^*^	0.06 (0.21)	−0.001 (0.98)
I am worried that I will have difficulty seeing the health care provider over telemedicine	0.11 (0.02)	−0.003 (0.95)	0.11 (0.01)^*^	0.06 (0.18)	0.02 (0.66)
I am worried about confidentiality of my private information being exchanged through telemedicine	0.04 (0.41)	0.09 (0.05)^*^	0.07 (0.12)	0.01 (0.76)	−0.07 (0.12)
I am worried about the continuity of care (i.e., I do not see the same provider every time)	0.03 (0.57)	0.10 (0.04)^*^	0.01 (0.80)	0.04 (0.39)	−0.003 (0.95)
I do not have the technology needed for telemedicine visits	0.04 (0.31)	0.11 (0.02)^*^	0.09 (0.04)^*^	0.09 (0.04)^*^	−0.12 (<0.001)^**^
I think it would take longer to have a visit over telemedicine than in person	0.05 (0.26)	0.19 (<0.001)^**^	0.05 (0.26)	0.07 (0.11)	−0.10 (0.02)^*^
I am worried about the quality of care I would receive	−0.02 (0.61)	−0.11 (0.01)^*^	0.01 (0.83)	0.01 (0.78)	0.05 (0.27)
I would most likely use telemedicine now because of the coronavirus (COVID-19)	−0.01 (0.85)	0.02 (0.74)	−0.04 (0.39)	−0.12 (<0.001)^**^	0.06 (0.15)

*p*-Value (significance), Pearson correlation factor.

^*^ *p*-Value <0.05.

^**^ *p*-Value <0.001.

There were no statistically significant correlations between demographic factors such as insurance status, gender, and race with our study's survey items. (See Table 1 for demographic information). However, there were positive correlations between age and concerns with confidentiality (*r* = 0.09, *p* = 0.05) and continuity of care (*r* = 0.96, *p* = 0.04) with their PCP. Meaning, as participants age increased, so did concerns of privacy and continuity of care with their PCP if using telemedicine services. (See Table 3 for correlation coefficients.) For example, in this study's population, participants with high educational attainment or higher income had increased access to technology and internet services.

## Discussion

This article sought to identify reasons that people had not used telemedicine before the pandemic. The results of this study demonstrate that participants perceived in-person care from their primary health care provider to be better than a visit done using telemedicine. Similar to past studies,^9^ these data show that PCPs are influential to their patients, and they can encourage future use and extol the benefits to patients. This is particularly true for vulnerable populations, who have concerns with privacy and continuity of care when utilizing telemedicine services. Results from a recent telemedicine study demonstrated that people who were new to the service adopted it because their primary care person was the provider, and the majority were satisfied with their visit(s).^5^

Additional results from this study also suggest that access to technology and the internet did not play a significant role in why these participants have not used telemedicine. This was somewhat surprising as past study has stated that this tends to be a major barrier.^10^ Therefore, we should focus on the other perceived barriers that keep people from accessing care in this way. One challenge may be that the older participants within our study were notably concerned with privacy issues related to telemedicine use and the continuity of care with their PCP. However, one study has demonstrated that as older adults use telemedicine, their concerns with privacy decrease.^11^ This finding shows that the uptake of telemedicine services and the increased use of these services among older adults may help ease their privacy concerns as they get more comfortable utilizing these services.^12^ Future research should seek to understand ways that PCPs' information sharing about confidentiality and privacy with their older patients impacts the patient's telemedicine uptake and ways to ensure that the use of telemedicine will not impact their accessibility with their primary care person.^13,14^ For example, studies could examine ways primary care offices and health systems can enhance support measures for older adults interested in telemedicine by providing written instructions on using these services for their telehealth visits and communicating in writing how their health information will be securely shared and stored.^15^ Another area of possible future research interest is at the system level; for example, having someone from the health system help set up individuals on the system before the visit to get them more comfortable with the equipment and answer any questions they might have.^16^

Although most of the survey participants reported they have the technology that is not a barrier of using telemedicine services, our results revealed that income and educational attainment do impact technology access. Specifically, participants with lower educational attainment and income had less access to quality internet or the technology needed to use telemedicine. Since the start of COVID-19, many health care providers and researchers are concerned that the increased use and push of telemedicine among patients may worsen health disparities among populations who have limited access to technology and internet services.^17–19^ Although the population of this study had the technical know-how and access to technology, there are many populations that have been forgotten. For example, those living in America's rural regions or those populations who have been historically marginalized have been disproportionately impacted by limited availability of technology and internet. Moreover, with much of society, including health care appointments being transitioned to online platforms during the pandemic,^5^ access to high-speed internet is an extreme necessity.^20^

Populations that disproportionately encounter these digital divide issues are individuals in lower income households, rural populations, and those a part of historically marginalized racial/ethnic groups.^18^ Past research demonstrates that individuals encounter three main barriers when assessing telemedicine: reliable internet coverage, absence of technology, and digital literacy.^19^ This digital divide problem is even more severe now that PCPs are advocating for telemedicine services. More recently, President Joe Biden's infrastructure plan passed, and it aims to improve access to broadband coverage to assist low-income individuals and other vulnerable populations.^21^ However, despite the passing of the trillion-dollar plan, the current health insurance aid through the Coronavirus Aid, Relief, and Economic Security act to cover telemedicine costs will soon come to an end on December 31, 2021.^22^ Thus, there is still a need for a heavier push on federal and local stakeholders to address these digital divide and health coverage issues; if not, the most vulnerable populations will be left out from getting the quality health care they deserve.

As with every study, there are limitations. This study's participants were predominately White, younger, highly educated, and wealthier than the general public. This limitation is due to the constraints of the study's convenience sampling. For example, our study's population was recruited through online methods; thus, our study's participants had access to technology that is not reflective of populations most at-risk for health disparities. Furthermore, studies should explore more diverse populations to examine whether differences in nonuse of telemedicine emerge. Previous research posits the high rates of unreadiness to utilize telemedicine services were found in populations such as people living in rural areas, those with lower levels of education and income, and among Black and Hispanic communities.^17,20^A second limitation of this study is that it relies on self-reported measures that may add bias to results. Although these limitations exist, this survey still helps us understand why some individuals are not utilizing telemedicine services and the role PCPs and health systems can play in encouraging the use of telemedicine.

## Conclusion

Since the start of the global pandemic, there has been an increased uptake of telemedicine services. Although the risks of COVID-19 will be mitigated, the use of telemedicine will remain salient. Therefore, characterizing the barriers of use is key, for this study, the main issue highlighted was individuals' having a strong relationship with their PCPs, and not a technological barrier. Therefore, having more opportunities to see a PCP over telemedicine and educating patients on its use would help to facilitate future use and comfort level. Although telemedicine is not ideal for all types of medical visits, the satisfaction that people have reported in telemedicine visits should level health systems and insurers to continue to allow and to consider keeping some form of the practice. As the world emerges from this pandemic, telemedicine will most likely remain an option for providing care, and as such, they will need to have continuous and varying communication with their patients about the opportunities and benefits of telemedicine.

## Authorship Contribution Statement

Bree E. Holtz devised this study. Robyn B. Adams drafted the article. Robyn B. Adams, Bree E. Holtz, and Victoria R. Nelson, completed the analyses. Robyn B. Adams, Bree E. Holtz, and Victoria R. Nelson, read and approved the final draft of the article.

## Abbreviations Used

PCP: primary care provider
SD: standard deviation
